# Filterability of Erythrocytes in Patients with COVID-19

**DOI:** 10.3390/biom12060782

**Published:** 2022-06-03

**Authors:** Dmitry S. Prudinnik, Elena I. Sinauridze, Soslan S. Shakhidzhanov, Elizaveta A. Bovt, Denis N. Protsenko, Alexander G. Rumyantsev, Fazoil I. Ataullakhanov

**Affiliations:** 1 Dmitry Rogachev National Medical Research Center of Pediatric Hematology, Oncology and Immunology, Ministry of Healthcare of Russian Federation, Samory Mashela Str., 1, GSP-7, 117198 Moscow, Russia; maddima285@gmail.com (D.S.P.); shakhidzhanov.s@yandex.ru (S.S.S.); ie.bovt.rv@gmail.com (E.A.B.); alexrum47@mail.ru (A.G.R.); 2 Center for Theoretical Problems of Physicochemical Pharmacology, Russian Academy of Sciences, Srednyaya Kalitnikovskaya Str., 30, 109029 Moscow, Russia; 3 City Clinical Hospital No. 40 Moscow Health Department, Kasatkin Str., 7, 129301 Moscow, Russia; drprotsenko@me.com; 4 Department of Anaesthesia and Critical Care, Pirogov Russian National Research Medical University, Ostrovityanov Str., 1, 117997 Moscow, Russia; 5 Department of Biophysics, Physics Faculty, Lomonosov Moscow State University, Leninskie Gory, 1, Build. 2, GSP-1, 119991 Moscow, Russia; 6 Moscow Institute of Physics and Technology, National Research University, Institutskiy Per., 9, 141701 Dolgoprudny, Russia; 7 Perelman School of Medicine, University of Pennsylvania, 3400 Civic Center Blvd., Philadelphia, PA 19104, USA

**Keywords:** COVID-19, erythrocyte, filterability, SpO_2_/FiO_2_ ratio, inflammation, dynamics of filterability alteration, additional oxygenation

## Abstract

For the first time, the influence of COVID-19 on blood microrheology was studied. For this, the method of filtering erythrocytes through filters with pores of 3.5 μm was used. Filterability was shown to significantly decrease with the increasing severity of the patient’s condition, as well as with a decrease in the ratio of hemoglobin oxygen saturation to the oxygen fraction in the inhaled air (SpO_2_/FiO_2_). The filterability of ≤ 0.65, or its fast decrease during treatment, were indicators of a poor prognosis. Filterability increased significantly with an increase in erythrocyte count, hematocrit and blood concentrations of hemoglobin, albumin, and total protein. The effect of these parameters on the erythrocyte filterability is directly opposite to their effect on blood macrorheology, where they all increase blood viscosity, worsening the erythrocyte deformability. The erythrocyte filterability decreased with increasing oxygen supply rate, especially in patients on mechanical ventilation, apparently not due to the oxygen supplied, but to the deterioration of the patients’ condition. Filterability significantly correlates with the C-reactive protein, which indicates that inflammation affects the blood microrheology in the capillaries. Thus, the filterability of erythrocytes is a good tool for studying the severity of the patient’s condition and his prognosis in COVID-19.

## 1. Introduction

Coronavirus disease COVID-19, caused by a new strain of coronavirus SARS-CoV-2 (severe acute respiratory syndrome coronavirus 2), was first detected in December 2019 in the People’s Republic of China in the city of Wuhan. Since then, the number of patients infected with SARS-CoV-2 worldwide has reached more than 500 million and continues to grow, accompanied by high mortality rates (to date, more than 6.2 million people have died) [1]. The causes of the severe course of the disease, along with others, are pathological disorders of the microvasculature with a bright alveolar-hemorrhagic syndrome (microangiopathy) [2], hypoxemia [3], as well as serious disorders of the blood coagulation system, which are clinically manifested in the form of thrombosis, including pulmonary embolism (PE), or thrombohemorrhagic syndrome [4,5,6,7]. One of the main causes of death with COVID-19 is progressive respiratory failure [8,9]. At the same time, one of the reasons for the decrease in the delivery of oxygen to organs and tissues may be the deterioration of blood rheology. Only a few articles have been published on the effects of COVID-19 on properties of erythrocytes (red blood cells, RBCs) and blood rheology [10,11,12,13]. In patients with COVID-19, important parameters, such as blood viscosity [10,13], the ability of RBCs to aggregate [10,13], deformability of erythrocytes [10,11,12,13], as well as their shape [11,12], size, and distribution of size and deformability [11], have been investigated. Blood viscosity and RBC aggregation have been shown to increase in COVID-19 patients. Nader E. et al. [13] found that these patients had a reduced hematocrit but an increased aggregation of RBCs. The authors associated this with an increase in fibrinogen levels in patients with COVID-19. Fibrinogen was also responsible for increasing blood coagulation in these patients, which was detected in this work by rotational thromboelastography (by increasing the clot firmness value (CFM) and reducing the coagulation time (CT)).

An important parameter, from the point of view of blood rheology, is the ability of RBCs to deform since the deterioration of deformability leads to a decrease in the ability of RBCs to pass through narrow capillaries and perform their gas transportation function. This, in turn, can exacerbate the acute respiratory failure often seen in COVID-19. The deformability of RBCs was investigated in all the above-mentioned works [10,11,12,13], but the data obtained were contradictory. In the works of Renoux C. et al. [10] and Kubankova M. et al. [11], a decrease in the deformability of RBCs in COVID-19 was found, while in the works of Piagnerelli M. et al. [12] and Nader E. et al. [13], no violations of the ability of RBCs to deform in such patients were found. In the works [10,11], the authors explain the decrease in the deformability of RBCs of patients with COVID-19 by the fact that the oxidative stress existing in patients affects erythrocytes, leading to changes in the lipid composition of the membrane, fragmentation of membrane proteins and metabolic disorders of erythrocytes described in the work [14]. 

It should be noted that the factors affecting the rheology of blood in large vessels with a diameter exceeding the diameter of the erythrocyte, and microrheology in vessels whose diameter is less than the diameter of the erythrocyte, are different. In vessels of large caliber, the shear stresses are very small, and the key role is played by blood viscosity, which increases with an increase in hematocrit, plasma concentrations of albumin, globulins, and fibrinogen, as well as with an increase in RBC aggregation [15,16]. A decrease in the ability of RBCs to deform also slightly increases the viscosity of the blood [17]. Thus, the effect of RBC deformability on blood rheology in large vessels is very small. Their main influence on blood rheology is carried out through the influence on the microrheology of blood in narrow capillaries. Here, the shear stress is in several tens of times more [18]. RBCs have to squeeze through these capillaries, which is impossible without the ability to deform. Microrheology can be affected by the viscosity of the cytoplasm of the RBC, the ratio of its surface area to volume (the higher it is, the better the cell’s ability to deform), the heterogeneity of RBCs by volume (the higher it is, the worse the overall deformability of the erythrocyte suspension), viscoelastic properties of the membrane, mean volume (MCV) and cell morphology [16]. RBCs in the blood always have a certain distribution in size and in the ability to deform. With a number of pathologies, a small (or even very small) fraction of cells may appear in the blood that are not able to pass through narrow capillaries. Even one such erythrocyte is enough to completely block the capillary. Despite the small proportion of such cells, under in vivo conditions they can have a strong effect on the total passage of RBCs through microcapillaries, worsening microrheology and causing hypoxemia. Although the presence of such cells will not affect the macrorheology of blood in large vessels, it is these cells that will determine the microrheology in the capillaries. Thus, it is important to choose a method for determining the deformability of RBCs, which would be sensitive to the presence in the blood of RBCs that are not able to pass through the capillaries. Measurement of RBC deformability in almost all previously published studies [10,12,13] was carried out by the method of ektacytometry, which is now considered the “gold standard” for measuring the deformability of RBCs. The exception is the work [11], in which the deformability of erythrocytes and other blood cells was assessed by the method of real-time deformability cytometry. The ektacytometer analyzes the diffraction pattern obtained for the studied suspension of cells, at different values of the applied shear stress. The method evaluates the average characteristic of RBC deformability in the population [19]. Due to the fact that the proportion of cells that are not able to pass through capillaries is usually very small, the method of ektacytometry is not sensitive to the presence of such cells. Kubankova M. et al. [11] examined the physical phenotypes (including mechanical properties) of five types of blood cells in patients with COVID-19 (erythrocytes, leukocytes, monocytes, neutrophils, and eosinophils). Size, deformability (by deviation of the shape of RBCs from normal discoid), as well as the distributions of cells by size and deformability, were measured. The study used real-time deformability cytometry, which is based on fixing images of cells in a sample of diluted (80 times) whole blood flowing through a microcamera located under a microscope. The transverse size of the channel through which the suspension of cells flows is 20 μm × 20 μm, which significantly exceeds the size of the RBC. Thus, cells flow through this channel freely (as in large vessels), and do not squeeze through it, as in the capillaries of the body. The size and deformability data of all cells are used to form dot-plots. On this plane, you can see not only the average values of the measured parameters, but also their distribution. The method registers the appearance of cells with reduced deformability but does not allow for assessing their ability to pass through narrow capillaries. The paper [11] analyzed blood samples from 17 COVID-19 patients in the intensive care unit, 14 patients 4 months after recovering from COVID-19, and 24 healthy donors. Patients with COVID-19 have been found to have a small fraction of RBCs with reduced size and deformability. In such patients, the distributions of RBCs by size and deformability significantly expand. The groups of healthy donors (control group) and recovered patients in most of the measured parameters practically did not differ, but the width of distribution by deformability of RBCs of recovered patients remained higher than in the control even 4 months after recovery. Unfortunately, the work [11] does not analyze the relationship of the measured indicators with the severity of the patient’s condition, as well as the change in the deformability of RBCs in dynamics during treatment.

As mentioned above, the RBC deformability affects, first of all, the microrheology of the blood, including due to the possible presence in the RBCs population of a small proportion of poorly deformable cells. The goal of our work was to study the ability of RBCs of patients with COVID-19 to pass through narrow capillaries. To achieve this, a method was used to measure the filterability of RBCs through the pores of a membrane filter with a diameter of 3–5 μm [20,21,22]. An important feature of this method is that the number of pores on the membrane through which RBCs are filtered is more than an order of magnitude less than the number of cells passing through it (approximately 2.50 × 10^7^ cells versus 6.11 × 10^5^ pores). Cells with poor filterability gradually clog the pores of the filter, reducing the overall rate of filterability. Thus, this method is sensitive to the presence in the suspension of studied RBCs of even a very small fraction of cells with poor filterability, which in vivo can have a strong effect on blood microrheology and lead to hypoxemia and hypoxia. 

To determine how the violation of the filterability of RBCs can be associated with the severity of the disease and its final outcome, the change in filterability during the course of the disease for a part of patients (n = 39) was investigated in dynamics. As a result, it was shown that the average filterability in the group of patients is reduced compared to the similar filterability in the group of healthy donors, and this decrease is greater the higher the severity of the disease. It is shown that the level of filterability ≤ 0.65 indicates a high probability of a poor prognosis. The change in filterability during the course of the disease was also different in the groups of patients who recovered and died after COVID-19. In the group of deceased, a decrease in filterability was most often observed throughout the hospital stay, while in the group of recovered patients, such a decrease was either absent at all or was very insignificant. Thus, a sufficiently high rate of decrease in RBCs filterability during the course of the disease may be a prognostic factor in the negative outcome of the disease.

Our analysis of correlations of filterability with a number of biochemical and hematological parameters showed that filterability increases with an increase in the number of erythrocytes, the hematocrit, and concentrations of hemoglobin, albumin, and total protein. This effect is directly opposite to the influence of these parameters on blood macrorheology, where an increase in all these factors leads to an increase in blood viscosity, which worsens macrorheology [15]. The presence of a negative correlation between the RBC filterability and the concentration of C-reactive protein suggests that inflammation may affect RBCs in patients with COVID-19.

## 2. Materials and Methods

### 2.1. Patients and Donors

The study included 149 non-consequent patients of the City Clinical Hospital No. 40 of the Moscow City Health Department diagnosed with COVID-19 who were admitted to the hospital from 29 July to 28 October 2020 and from 11 May to 30 July 2021, as well as 46 healthy donors as a control group. The diagnosis was confirmed by polymerase chain reaction (PCR), enzyme-linked immunosorbent assay (ELISA) and/or computed tomography of the chest cavity (CT). All patients were over 18 years of age. The criteria for exclusion were violations in the blood coagulation system, pregnancy, and transfer from another hospital. Patients had various comorbidities, such as cancer (n = 14 (9.4%)), diabetes (n = 49 (32.9%)), cardiovascular disease (n = 112 (75.2%)), kidney disease (initially observed in 31 patients (20.8%), and in 25 patients (16.8%) kidney failure was observed as a complication during the disease), liver disease (initially observed in 2 patients (1.3%), and in 3 patients (2.0%) liver failure was observed as a complication during the disease), and atherosclerosis (n = 36 (24.2%)). For each patient, clinical indicators were collected, which included routine biochemical and hematological parameters, analysis of the state of the blood coagulation system and measurement of RBCs filterability. Data on the demographic composition and some characteristics of patients’ condition are presented in Table 1 and Table S1 in Supplementary Materials. The control group included 46 apparently healthy donors (men—65.3%, women—34.7%; average age 36 (19 to 75 years) without COVID-19. For the control group, only the filterability of RBCs was measured.

### 2.2. Ethical Statement

The study was conducted according to the guidelines of the Declaration of Helsinki and approved by the Independent Ethical Committee of Dmitriy Rogachev National Medical Research Center of Pediatric Hematology, Oncology, and Immunology, Ministry of Healthcare of Russia, Moscow (Permit Number: 3e/1-20 from 19 May 2020). Informed consent was obtained from all subjects involved in the study. 

### 2.3. Materials

All reagents for buffer preparation were purchased from Sigma-Aldrich (St. Louis, MO, USA). Polyethylenterephthalat membrane filters with a pore diameter of 3.5 μm were from the Joint Institute for Nuclear Research (Dubna, Moscow Region, Russia). All standard laboratory diagnostic tests were carried out by standard methods with standard sets of reagents used in the hospital.

### 2.4. Preparation of Blood Samples for Filterability Measurement

Blood from healthy donors and patients (3.6 mL) was collected into standard UNIVAC vacuum tubes (Eiliton LLC, Dubna, Russia) with 3.2% (0.109 M) solution of trisodium citrate dihydrate (the citrate/blood ratio was 1/9). RBCs were separated by centrifugation at 1000× *g* for 10 min using medical centrifuge CM-6M (ELMI, Riga, Latvia). Plasma and buffy-coat were removed. RBCs were washed one time in normal saline, followed by centrifugation for 10 min at 1000× *g*. Saline-washed RBCs (400 μL) were mixed with 1600 μL of Tyrode’s buffer (135 mM NaCl, 4 mM KCl, 0.33 mM NaH_2_PO_4_, 1 mM MgCl_2_, 11 mM glucose, 2.5 mM CaCl_2_, 10 mM HEPES, and 1 mg/mL of bovine serum albumin, pH 7.4), and washed by centrifugation (8 min, 1000× *g*) using an Eppendorf centrifuge (Hamburg, Germany). Suspension of washed RBCs (240 μL) taken from the bottom of the test tube (with a hematocrit of approximately 95%) was mixed with 120 μL of Tyrode’s buffer (2:1). To the 32 μL of the resulting suspension, 1888 μL of the Tyrode’s buffer were added (1:59). The resulting suspension, which was used to measure the filterability of RBCs, had a hematocrit of 1 ± 0.05%.

### 2.5. Measurement of Erythrocyte Filterability

The filterability of RBCs was determined by the rate of their passage through artificial membrane filters using the IDA-01 filterometer [23]. This device measures with an accuracy of 0.1 s the flow time of 250 μL of the buffer or studied RBC suspension through a membrane filter with pores with a diameter of 3–5 μm using a sensor mounted in a lid that closes the reservoir filled with buffer solution. The sensor is a system of three needle electrodes of different lengths, immersed to different depths and associated with an electronic time meter. The measurement of time starts at the beginning of the fluid outflow and stops after the outflow of 250 μL.

For measurements, a polyethylenterephthalat filter with pores with a diameter of 3.5 μm and a pore length of 10 μm (Joint Institute for Nuclear Research, Dubna, Moscow Region, Russia) was used, on which 570 μL of Tyrode’s buffer or the studied suspension of RBCs (with Hct 1%) was placed. Filterability (F) was characterized by the relative rate of suspension passing through the filter, which was defined as the ratio of the passing time of 250 μL of the buffer to the analogous time for 250 μL of cell suspension:

F = tb/ts

where tb and ts are the time of flow through the filter of the buffer or the studied cell suspension, respectively. The faster the cell suspension passes through the filter (i.e., the shorter the time ts), the higher the filterability of these cells. In total, 342 measurements of RBCs filterability were made in patients. In 39 out of 149 patients, the erythrocyte filterability index was measured in dynamics 3 or more times during hospitalization, in the remaining 110 patients, measurements were carried out 1 or 2 times.

### 2.6. Measurement of the Dependence of Erythrocyte Filterability on the Severity of the Patient’s Condition

All patients (n = 149) were divided into the following groups depending on the severity of the condition: Control (healthy donors (n = 46)), Moderate (moderate patients (n = 21)), Severe (severe patients (n = 103)) and Critical (very severe patients (n = 25)). When dividing into groups, many indicators were taken into account: the ratio of the percentage of oxygen saturation to the oxygen fraction in the inhalation mixture (SpO_2_/FiO_2_), the results of clinical and biochemical blood tests, as well as the values of scores obtained for the patient on the SOFA [24], NEWS [25], or APACHE [26] scales. The first of them helps to identify the organ failure present in the patient and is based on the data of a number of biochemical parameters, the second is usually used to quickly assess the severity of the patient’s condition. To assess the severity of the condition, it uses indicators, such as SpO_2_, respiratory rate, temperature, systolic pressure, heart rate, state of consciousness, as well as an assessment of the need for additional oxygen. In addition, the degree of lung damage assessed using computed tomography was taken into account. The APACHE scale is used to assess the likelihood of lethal outcome in the intensive care unit. It includes data on the patient’s state of health before admission to the hospital, as well as 34 physiological indicators that allow us to assess the severity of the disease upon admission to the hospital, due to the state of the main body systems (neurological, cardiovascular, respiratory, gastrointestinal, renal, and hematological). All filterability measurements were compared with the patient’s severity at the time of measurement. In order to assign each patient only one point in this correlation, his RBCs filterability values were averaged if he had several filterability measurements but remained in the same disease severity group. If during treatment the degree of severity in the patient changed, then only the point corresponding to a higher degree of severity was taken into account. Figure S1 in Supplementary Materials shows the same correlation, which contains all the points measured for each patient.

### 2.7. Measurement of the Dependence of Erythrocyte Filterability on the Level and Type of Additional Oxygenation

In the study of the correlation of the level of filterability with the level of supplemental oxygen supply, all patients were divided into the following groups: Control (group of healthy donors, breathing with atmospheric air, oxygen content of 21%), Independent (group of patients on independent breathing with atmospheric air, oxygen content of 21%), Low-flow (group of patients on low-flow oxygenation, oxygen fraction in inhaled air 24–35%), High-flow (patients receiving high-flow oxygenation, oxygen fraction in inhaled air 40–100%) and MV (patients on invasive mechanical ventilation, oxygen fraction in inhaled air 25–100%). For all these patients, the filterability of the RBCs was measured. If the patient had several measurements of filterability but remained in the same oxygen supply group, his filterability values were averaged. If the patient during treatment moved from one group to another, then only his indications in the group with higher oxygenation (or MV) were presented. Figure S3 (Supplementary Materials) shows a similar dependence with all points measured for each patient. 

### 2.8. Data Analysis

The data were processed and analyzed using the program RStudio 2021.09.0 + 351 ‘Ghost Orchid’ Release. The average values for each of the biochemical, hematological and coagulation parameters presented in Table 1 and Table S1 were estimated for all measurements of this parameter as the median and region (from 25 to 75 percentiles). The significance of the difference between groups of patients was assessed using Kruskal–Wallis H-test and post-hoc Dunn’s test with Bonferroni correction. The differences were considered significant at *p* < 0.05. The *p* levels obtained in calculating the difference between the groups are shown in the figures (from *p* < 0.05 to *p* < 0.0001). To search for correlations between the filterability of RBCs and the results of routine laboratory tests, values obtained on the same day were used. For all correlations, a linear approximation was used, which was characterized by the Spearman correlation coefficient (R). The existence of a statistically significant relationship between filterability groups and mortality was proved by the Fischer’s exact test with Monte Carlo simulations (*p* = 0.0004998).

## 3. Results

### 3.1. The Dependence of Erythrocyte Filtration on the Severity of the Patient’s Condition

**Figure 1:**
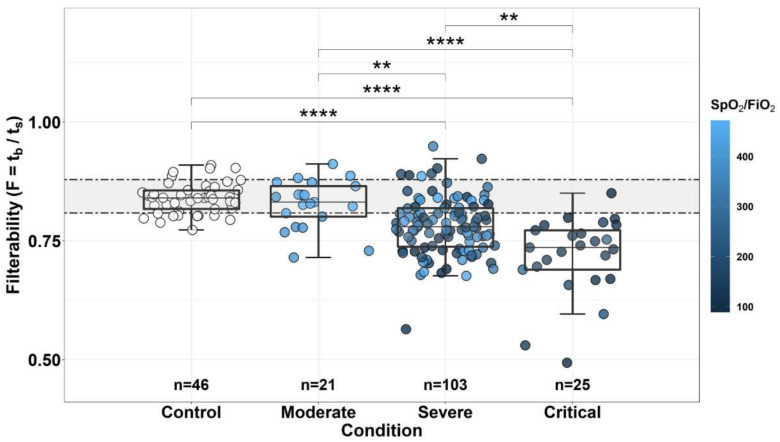
Dependence of erythrocyte filterability on the severity of the patient’s condition. Control—control group, Moderate—a condition of moderate severity, Severe—a serious condition, Critical—a condition of extreme severity. In addition, each represented point is automatically colored according to the SpO_2_/FiO_2_ ratio for given measurement (see the color scale to the right of the figure). The significance of the differences between the groups was calculated using the Kruskal–Wallis H-test and post-hoc Dunn’s test with Bonferroni correction. Horizontal dotted lines show the boundaries of normal filterability values. The box sizes correspond to the range comprising from 25 to 75 percentiles of all measured values. Medians are indicated by horizontal lines, and the length of the whiskers corresponds to the 1.5 interquartile range. The *p* values for the significance of the differences are shown in the figure: ** *p* < 0.01 and **** *p* < 0.0001.

The obtained correlation between the severity of the patients’ condition and the level of filterability of their RBCs is presented in Figure 1. When dividing patients into groups with different severity of the condition (see Materials and Methods, Section 2.6), the following groups were distinguished: control (healthy donors, n = 46), patients in moderate condition (n = 21), patients in severe condition (n = 103) and patients in critical condition (n = 25). Since the RBC filterability parameter does not obey the normal distribution (Shapiro–Wilk test: W = 0.95389, *p* = 5.913 × 10^−6^), the Kruskal–Wallis criterion was used to determine the reliability of the differences between the groups. When comparing filterability for groups of different severity, this criterion showed significant differences (Kruskal–Wallis χ^2^ = 6 3.584, df = 3, *p* = 1.007 × 10^−13^). For a pair comparison of groups, Kruskal–Wallis test and post-hoc Dunn’s test with Bonferroni correction were used. All the groups studied, with the exception of the Control and Moderate groups, differed significantly in the level of erythrocyte filterability, which decreased with increasing severity of the condition. 

### 3.2. The Dependence of the Filterability of Erythrocytes of Patients on the Level of the SpO_2_/FiO_2_ Ratio

**Figure 2:**
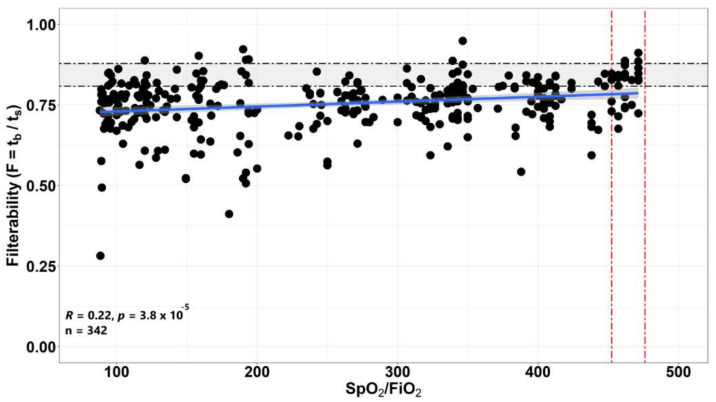
Correlation of RBC filterability in patients with COVID-19 with the value of the SpO_2_/FiO_2_ ratio, where SpO_2_ in each case is directly measured by a pulse oximeter, and FiO_2_ for each point corresponds to the proportion of oxygen in the inhaled mixture (for atmospheric air FiO_2_ = 0.21, the proportion of oxygen in the inhaled air at different oxygen flow rates was calculated by the standard method [27]). The gray area represents the confidence interval. Horizontal dotted lines show the boundaries of normal filterability values.

Since the severity of the condition in COVID-19 can largely be attributed to insufficient oxygenation, an analysis of the correlation of erythrocyte filterability with the level of the SpO_2_/FiO_2_ ratio, which is the ratio of the percentage of oxyhemoglobin in the blood to the proportion of oxygen in the inhaled mixture, was carried out for all available measurements. The higher the percentage of hemoglobin bound to oxygen at a certain proportion of oxygen in the respiratory mixture, the more efficiently this oxygen is used. In donors, SpO_2_ and FiO_2_ values were not determined, but under normal conditions the SpO_2_/FiO_2_ ratio should be >452. For all available measurements, the correlation of the value of this ratio with the value of filterability is constructed (Figure 2). The obtained correlation reliably showed an increase in filterability with an increase in the SpO_2_/FiO_2_ ratio (R = 0.22, *p* = 3.8 × 10^−5^).

The dependence of the severity of the patient’s condition on the value of the SPO_2_/FiO_2_ ratio can also be seen in Figure 1, where all points were automatically colored according to the value of this ratio (see the color scale to the right of the figure). It is clearly seen that if in a group of patients in a state of Moderate severity, the value of this ratio does not fall below 400, with an increase in the severity of the condition (in Severe and, especially, Critical group), the number of patients with a greatly reduced SpO_2_/FiO_2_ ratio increases to about 50% and 100%, respectively. For the Control group we did not measure this ratio (all the points in this group were not colored), but its value of >450 was considered normal. The correlation of the SpO_2_/FiO_2_ ratio with the patient’s condition is also presented in Figure S2, which shows that the differences between these groups are significant. Thus, the value of this ratio correlates well with the severity of the patient’s condition. 

### 3.3. Effect of Erythrocyte Filterability of COVID-19 Patients on Disease Outcome

**Figure 3:**
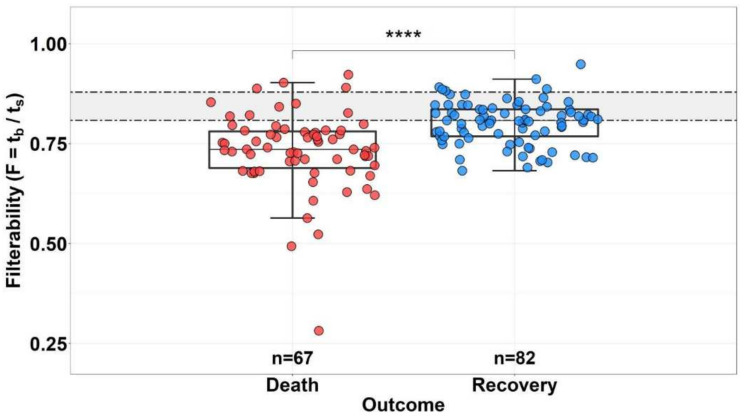
Distribution of RBC filterability in the group of deceased (n = 67) and survivor (n = 82) patients with COVID-19. Horizontal dotted lines indicate the boundaries of normal filterability values. **** The difference between the groups is significant (*p* < 0.0001).

To trace the effect of decreased filterability on patient deterioration, we first analyzed the outcome for all patients depending on the filterability of their RBCs (for patients in whom filterability was measured in dynamics, only the most recent measurement was taken into account) (Figure 3). On average, the filterability (F) was significantly higher in the group of surviving patients (median filterability values were 0.81 and 0.74 in the group of surviving and deceased patients, respectively).

**Figure 4:**
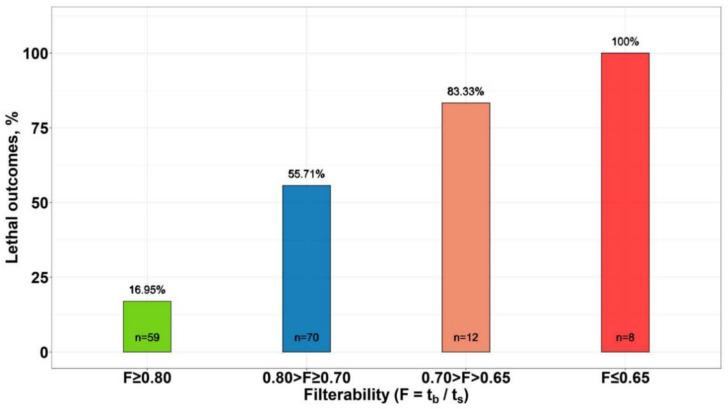
The percentage of deaths in groups of patients with different filterability of erythrocytes (F). The filterability levels in the groups were equal to: F ≥ 0.80; 0.80 > F ≥ 0.70; 0.70 > F > 0.65 and F ≤ 0.65. The figure shows the number of patients and the percentage of mortality in each group. Existence of a statistically significant relationship between filterability groups and mortality was proved by Fisher’s exact test with Monte Carlo simulation (*p* = 0.0004998).

For a more detailed analysis of the dependence of the mortality rate on the value of RBC filterability, all patients were divided by the level of filterability into the following groups: F ≥ 0.80; 0.80 > F ≥ 0.70, 0.70 > F > 0.65 and F ≤ 0.65. The percentage of lethal outcomes in each of these groups is presented in Figure 4. The number of deaths increased with a decrease in the level of filterability of RBCs, and in patients with F ≤ 0.65, the outcome in 100% of cases was fatal.

### 3.4. Study of Changes in Erythrocyte Filterability in Patients with COVID-19 in Dynamics

**Figure 5:**
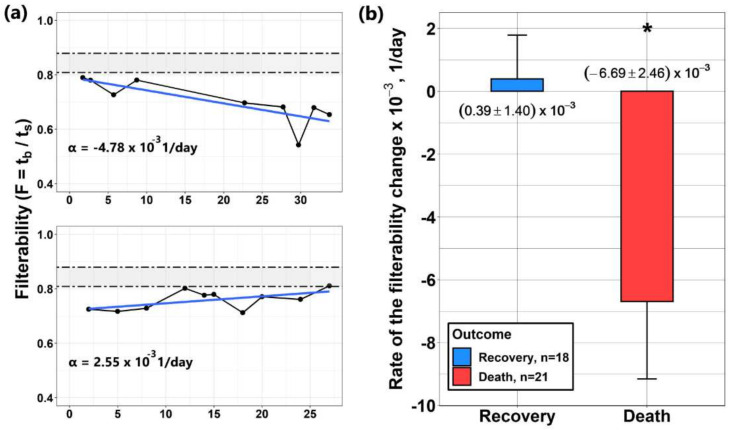
Dynamics of changes in the filterability (F) of erythrocytes in COVID-19 patients. (**a**) Typical examples of the dynamics of changes in the filterability of erythrocytes in two patients, showing that during treatment F can both increase and decrease. Dotted lines indicate the boundaries of normal filterability values. The rates of changes in filterability (α) for each of the patients are given. (**b**) Averaged rates of change in filterability in groups of surviving and deceased patients. The average values and standard errors of the mean (SEM) are presented. * The difference between the groups is significant (ANOVA, *p* < 0.05).

To study how the change in RBC filterability during COVID-19 treatment is related to the outcome of the disease, the RBC filterability in dynamics was tested for 39 patients. For each of them, F was measured 3 to 13 times during a hospital stay. In each case, plots of the dependence of filterability on time were constructed, which were approximated by linear dependencies. Figure 5a shows typical examples of the resulting dependencies.

The initial value of erythrocyte filterability in almost all patients was below normal. A rate of filterability change for each of the patients was defined as the slope of the approximating line. The sign of this slope determined the direction of change in filterability (an increase or decrease with time). The rates of change in F for most of the patients studied did not change very significantly, but the averaging of these rates for groups of surviving and deceased patients showed a significant difference in these rates (Figure 5b). Thus, if in the group of surviving patients (n = 18) F increased slightly (the average rate of F change was α = +(0.39 ± 1.40) × 10^−3^ 1/day), then in the group of deceased patients (n = 21), the filterability on average decreased at a rate of α = (−6.69 ± 2.46) × 10^−3^ 1/day.

As mentioned above, the changes in filterability for most of the patients studied were very small, and often weak changes in filterability were not directly related to the disease, i.e., weak changes in filterability in both directions (decrease or increase) could be observed in both groups. At the same time, with a sufficiently high rate of decrease in filterability, the outcome, as a rule, was unfavorable. Thus, it can be concluded that a relatively high rate of decrease in the filterability of RBCs during treatment may be a sign of a negative outcome of the disease. This correlates with the above conclusion that a decrease in filterability below 0.65 is a prognostic indicator of a poor outcome.

### 3.5. Dependence of the Erythrocyte Filterability on the Level and Method of Additional Oxygenation

The process of oxygenation of body tissues, in which RBCs play a key role, takes place in the capillaries of the microvasculature. Reduced oxygenation in patients diagnosed with COVID-19 is compensated by an increase in the level of oxygen supplied to patients, so it was interesting to investigate the dependence of the ability of erythrocytes to filter on the level and type of oxygen supply.

**Figure 6:**
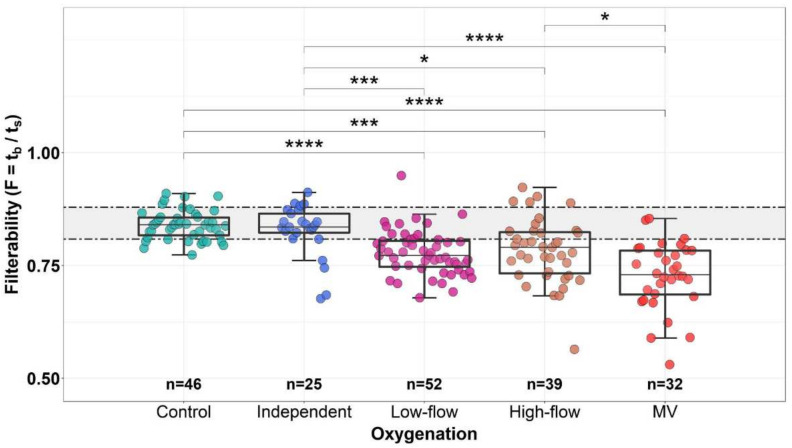
Dependence of the filterability of erythrocytes on the rate and mechanism of oxygen supply. Control (a control group of healthy donors) and Independent (patients without additional oxygen, who breathe with atmospheric air on their own)—oxygen fraction is 21%; Low-flow (patients on low-flow oxygenation)—the fraction of oxygen in the inhaled air is 24–35%; High-flow (patients on high-flow oxygenation)—the fraction of oxygen in the inhaled air is 40–100%; MV (patients on invasive mechanical ventilation)—the fraction of oxygen in the inhaled air can be 25–100%. The significance of differences between groups was calculated by the Kruskal–Wallis H-test and post-hoc Dunn’s test with Bonferroni correction. Horizontal dotted lines show the boundaries of normal filterability values. The box sizes correspond to the range comprising from 25 to 75 percentiles of all measured values, and the length of the whiskers corresponds to the 1.5 interquartile range. The *p* values of the significance of the differences between the groups are shown in the figure: * *p* < 0.05; *** *p* < 0.001 and **** *p* < 0.0001.

For this analysis, all patients were divided into the following groups (see Materials and Methods, Section 2.7): Control (healthy donor group, n = 46), Independent (group of patients on independent breathing with atmospheric air, n = 25), Low-flow (group of patients on low-flow oxygenation, n = 52), High-flow (patients receiving high-flow oxygenation, n = 39), and MV (patients on invasive mechanical ventilation, n = 32). One patient on extracorporeal membrane oxygenation (ECMO) was excluded from consideration. Filterability was measured for all these patients. The results obtained are presented in Figure 6. The median values of filterability obtained for each of the groups were: Control—0.84, Independent—0.84, Low-flow—0.77, High-flow—0.79, and MV—0.73. The filterability of erythrocytes of patients who did not receive supplemental oxygen and healthy donors did not differ significantly. Both of these groups breathed independently with atmospheric air (oxygen fraction 21%). The filterability of RBCs in the Low-flow and High-flow groups also did not differ significantly, despite the fact that the levels of oxygen supplied to patients in these groups differed significantly (24–35% vs. 40–100%, respectively). At the same time, the filterability was significantly reduced relative to the control group in the groups that received low-flow and high-flow oxygenation, as well as in patients who underwent mechanical ventilation. The lowest filterability of RBCs was noted in the group of patients on mechanical ventilation. At the same time, in each conditional group, distinguished by the type of oxygen supply, there were cases of low filterability of erythrocytes (F ≤ 0.65).

A similar correlation, but taking into account all repeating points measured for each patient, is presented in Figure S3 in Supplementary Materials. The consideration of additional points did not fundamentally change the results presented in Figure 6.

In addition to the correlation of filterability with the level of supplied oxygen, we decided to see how the values of the SpO_2_/FiO_2_ ratio are distributed into groups, which differ in the level of additional oxygenation. The lowest values of this ratio were observed in the High-flow and MV groups (Figure S3 in Supplementary Materials), in which the filterability was lower than in the control group, as well as in the Independent and Low-flow groups. Comparison of the Low-flow and High-flow groups showed that they do not differ significantly in terms of filterability; however, despite the fact that the High-flow group received more additional oxygen, in this group (as well as in the MV group) there were many more points with the greatly reduced SpO_2_/FiO_2_ ratio. We believe that the observed trend towards a decrease in filterability with an increase in additional oxygenation (or switching to MV) is not due to the effect of the oxygen directly on the filterability of RBCs, but to the fact that patients who need more additional oxygenation tend to have a more severe condition. However, as was shown above, with the deterioration of the condition, both the filterability of RBCs and the value of the SpO_2_/FiO_2_ ratio decrease.

### 3.6. Correlations of Erythrocyte Filterability with Routine Laboratory Tests 

**Figure 7:**
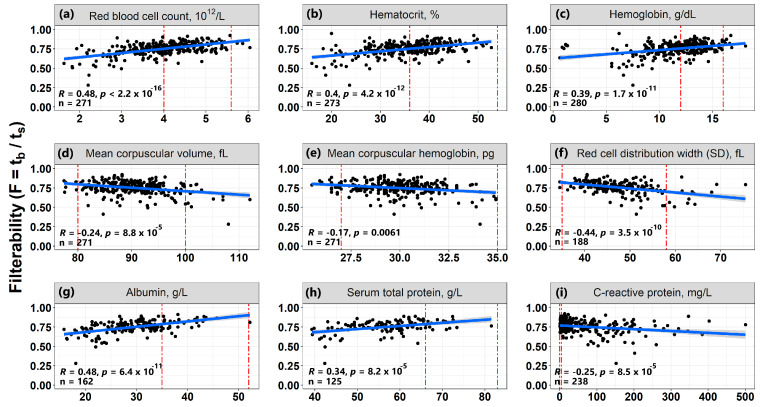
Correlations of some laboratory parameters with filterability (F) of RBCs from patients with COVID-19. Correlations F with the following parameters are presented: (**a**) Red blood cells count; (**b**) Hematocrit; (**c**) Hemoglobin; (**d**) Mean corpuscular volume; (**e**) Mean corpuscular hemoglobin; (**f**) RBCs distribution width by volume (as SD of average value); (**g**) Albumin; (**h**) Serum total protein; and (**i**) C-reactive protein. For all correlations, a linear approximation was used, which was characterized by the Spearman correlation coefficient (R). Gray ranges are confidence intervals of approximation line. The vertical red lines show the normal range for each of the parameters.

To determine which parameters primarily affect the filterability of RBCs of patients with COVID-19, correlations of filterability values with more than 40 different laboratory tests were investigated. Strong correlations (|R| > 0.5) were not found. The most pronounced correlations are presented in Figure 7. All obtained average values of various biochemical, hematological and coagulation indicators measured in the blood of COVID-19 patients are presented in Table 1 and Table S1 in the Supplementary Materials. The strongest positive correlations were found with the number of erythrocytes (RBC, R = 0.48), hematocrit (Hct, R = 0.4) and hemoglobin (Hb, R = 0.39), i.e., with parameters characterizing the general state of the erythroid cells (Figure 7a–c). Filterability also increased with increasing concentrations of albumin (R = 0.48) and total blood protein (R = 0.34) (Figure 7g,h).

Significant negative correlations were observed for the dependence of filterability on the distribution width of erythrocytes by volume (RDW-SD, R = −0.44), the average volume of an erythrocyte (MCV, R = −0.24), on the average content of hemoglobin in an erythrocyte (MCH, R = −0.17) (Figure 7d–f), as well as on the concentration of C-reactive protein (R = −0.25) (Figure 7i).

### 3.7. The Relationship of Erythrocyte Filterability with Inflammatory Processes

**Figure 8:**
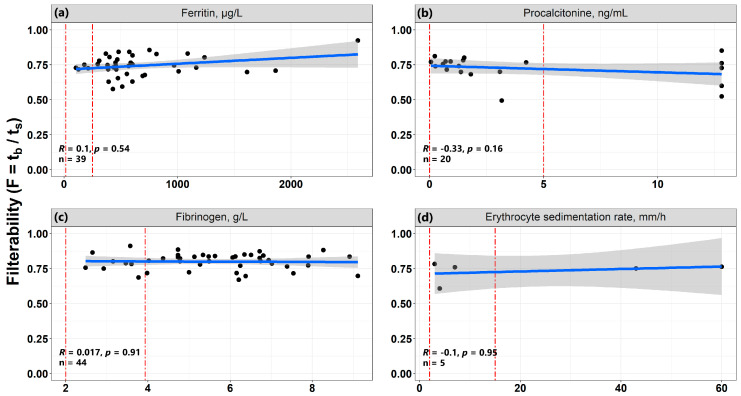
Correlations of RBC filterability in patients with COVID-19 with some markers of inflammation: (**a**) Ferritin (n = 39); (**b**) Procalcitonin (n = 20); (**c**) Fibrinogen (n = 44); and (**d**) Erythrocyte sedimentation rate (n = 5). The gray areas are the confidence intervals of the approximating straight line. The vertical red lines show the normal range for each of the parameters.

In order to trace a possible relationship between a decrease in the filterability of RBCs and the presence of an inflammatory process, such indicators as the level of C-reactive protein, fibrinogen, procalcitonin, ferritin, and erythrocyte sedimentation rate were analyzed. Among inflammatory markers, a weak significant negative relationship with the filterability parameter (R = −0.25, *p* = 8.5 × 10^−5^) was shown only by C-reactive protein, which is a protein of the acute phase of inflammation (Figure 7i). The higher the level of C-reactive protein (i.e., the higher the level of inflammation), the lower was the observed RBC filterability. Correlations with RBC filterability of several more parameters that are associated with the state of inflammation are presented in Figure 8. These are ferritin (R = 0.1, *p* = 0.54), procalcitonin (R = −0.33, *p* = 0.16), fibrinogen (R = 0.017, *p* = 0.91) and erythrocyte sedimentation rate (R = 0.1, *p* = 0.95) (Figure 8a–d, respectively). None of these parameters significantly correlated with filterability. 

## 4. Discussion

For the successful implementation of the gas transport function, erythrocytes must be able to pass through all the vessels of the body, including the smallest capillaries. Since the diameter of these capillaries is smaller than the size of the erythrocyte, in order to overcome them, the erythrocyte must be able to deform quite easily. The main effect of RBCs deformability on blood rheology is not related to their effect on its macrorheology in large vessels, where shear stresses are small and cells move freely through the vessels, but with their effect on microrheology in capillaries, where shear stresses are much higher. It is known that in various pathologies, the fraction of erythrocytes can occur in the blood, where erythrocytes have impaired deformability and are not able to pass through capillaries. This fraction may be insignificant in size, but very important from the point of view of blood microrheology. In an in vivo situation, one such erythrocyte can disconnect an entire capillary from the bloodstream, blocking it. Therefore, in order to correctly assess the impact of erythrocytes of COVID-19 patients on blood microrheology, we chose a method that is sensitive to the presence of such small fractions of cells with altered deformability—the method of filtering erythrocytes through artificial filters with pores slightly smaller than the size of the erythrocyte (3.5 μm). 

The main results obtained in this study are as follows:

1. The filterability of RBCs from patients with COVID-19 is reduced compared to normal RBCs. This decrease is all the more pronounced the more severe the patient’s condition.
2. The patient’s condition is also significantly correlated with the SpO_2_/FiO_2_ ratio.
3. The RBCs filterability (including when measured in dynamics during treatment) can be used not only as an indicator of the patient’s condition, but also as a prognostic indicator of the outcome of the disease.
4. The filterability of erythrocytes significantly increases with an increase in the blood of the number of erythrocytes, hematocrit, as well as concentrations of hemoglobin, albumin, and total protein. Such an effect is opposite to the effect of these parameters on blood macrorheology, since all of them increase blood viscosity, which worsens its macrorheology.
5. The existing inflammatory process may affect the properties of erythrocytes of patients with COVID-19.

With COVID-19 disease, a rather strong inflammatory process can occur in the body, which, of course, should affect both the state of the patient’s vessels and his RBCs, including their ability to deform. Currently, there are very few studies on the impact of COVID-19 on blood rheology and on the properties of RBCs, and none of them assess the ability of erythrocytes to pass through narrow capillaries 

In the present work, it was shown that the value of RBCs filterability in COVID-19 decreases. The decrease is more pronounced if the patient is in a more severe condition (Figure 1). In groups of patients in severe (Severe) and very severe (Critical) condition, several cases of extremely low filterability were observed (Figure 1), which was previously also noted in the study of Kubankova et al. [11]. In parallel, it was shown that the severity of the patient’s condition correlates with the value of the SpO_2_/FiO_2_ ratio, which characterizes the efficiency of the use of oxygen entering the body (Figure 1, Figure 2 and Figure S2). The lower this ratio, the more severe the patient’s condition. Since filterability also deteriorates with the deterioration of the patient’s condition, it can be assumed that the decrease in the efficiency of oxygenation observed with the deterioration of the patient’s condition may be due to a decrease in the filterability of his erythrocytes and may in some cases be the cause of this deterioration.

We showed that the patient’s condition is directly related to the filterability of his RBCs. Thus, the average filterability of erythrocytes in the group of surviving patients (F = 0.81) was significantly higher than in the group of deceased patients (F = 0.74) (Figure 3). A more detailed study showed that mortality increased as the filterability of patients’ RBCs decreased, and at filterability ≤ 0.65, mortality reached 100%. Thus, it can be concluded that although the cause of death in COVID-19 is not necessarily a decrease in erythrocyte filterability, when the filterability is reduced sufficiently (at F ≤ 0.65), this may be a factor indicating a poor prognosis. 

When studying the dynamics of changes in filterability during treatment, it was shown that for most of the studied patients, such changes were very insignificant, and often the direction of change in filterability was not directly related to the outcome of the disease. Weak changes in filterability, both in the direction of decrease and in the direction of its increase, were observed both in one and in the other group. At the same time, with a sufficiently high rate of filterability decrease, the outcome, as a rule, was unfavorable. Thus, although changes in filterability are very small in some cases, it can be concluded that a relatively high rate of decrease in filterability of RBCs during treatment may be a sign of a negative outcome of the disease. This correlates with the conclusion above that a drop in filterability below 0.65 is a predictor of poor outcome.

It was interesting to investigate how the level and type of supplemental oxygen supply may affect the change in the filterability of patients’ RBCs. Despite the fact that in Figure 6 there is a tendency to reduce filterability with an increase in the supply of oxygen, a good correlation is not observed here. The obtained result does not directly confirm the influence of the oxygen level directly on the filterability, because most likely, filterability here does not correlate with the level of oxygen supplied, but with the condition of the patient. The worse this condition is, the more likely the patient needs supplemental oxygen.

When studying the correlations of filterability with various laboratory indicators, it was shown that the filterability of RBCs increases significantly with an increase in the number of RBCs, hematocrit and the concentrations of hemoglobin, albumin, and total protein in the blood (Figure 7). It is interesting that these correlations are directly opposite to the correlations of these parameters with blood macrorheology in large vessels. An increase in the number of RBCs in the blood, its hematocrit and hemoglobin concentration increase blood viscosity, which leads to a deterioration in its macrorheology in large vessels [15,16,17]. On the other hand, a higher count of RBCs, Hct and Hb should be observed in the blood of patients whose RBCs were not subjected to strong influences and destruction as a result of the pathological process. Thus, these RBCs suffered little during the disease, and the less the patient’s RBCs were affected, the higher should be their filterability through narrow capillaries.

The increase in filterability with an increase in albumin concentration is in good agreement with the known fact that albumin contributes to the restoration of the normal shape of RBCs and a decrease in the number of echinocytes in suspension [28], which improves cell morphology and their filterability. At the same time, in the same work [28], it was noted that the average indicators of RBC deformability, measured using ektacytometry, did not improve in the presence of albumin.

Negative correlations of RBC filterability were observed with indicators, such as the width of the distribution of RBCs by volume (RDW-SD), the average volume of RBCs (MCV), and mean erythrocyte Hb content. An increase in RDW-SD indicates that cells of different volumes are present in the blood, including a small subpopulation of cells that cannot be filtered through the pores of the membrane filter. As mentioned above, the presence of even a small subpopulation of such cells significantly reduces the overall filterability of the erythrocyte suspension and can have a negative effect on the microrheology of RBCs in capillaries in vivo. The same applies to cells with elevated MCV. Increasing the mean Hb content of the RBCs can make the intracellular contents more viscous, which should reduce their ability to pass through narrow capillaries.

A significant decrease in the filterability of RBCs was also observed with an increase in the concentration of C-reactive protein, which is a protein of the acute phase of inflammation and is considered its marker. However, other indicators that may be associated with inflammation did not show reliable correlations with the filterability of erythrocytes. 

To date, it has already been shown that a sharp rise in the level of ferritin (one of the proteins of the acute phase of inflammation) in the blood serum to values > 1000–1500 μg/L is a prognostic sign of lethal outcome in COVID-19 [29,30]. Ferritin level correlates with markers of cell damage and oxidative stress, in particular the formation of hydroxyl radicals, and with disease severity. The main reason for the negative impact of ferritin on the condition of patients with COVID-19, the authors of the work consider not iron metabolism disorders and not the hemotoxic effect of the virus, but the fact that during inflammation the concentration of ferritin increases (the secretion of ferritin by macrophages increases and it is released from damaged cells). As a result, there is an increase in the production of pro-inflammatory factors (including the nuclear factor kB) by liver cells, and a cascade of reactions is triggered, leading to a cytokine storm, which is one of the main causes of death in COVID-19. Our data show that with an increase in the concentration of ferritin in the blood serum, the filterability of RBCs increases slightly (Figure 8a). At first glance, this seems strange, but we can explain this by the fact that during inflammation, some of the cells (the weakest and most defective) are destroyed. This increases the level of ferritin in the blood; however, the remaining cells are closer in their mechanical properties to the original unchanged RBCs, so the filterability of such cells may increase compared to a suspension that contains a proportion of partially defective cells.

The correlation of RBC filterability with procalcitonin levels is shown in Figure 8b. The concentration of this compound should increase with increased inflammation, but it is known that procalcitonin changes significantly only in the case of bacterial, but not viral, infection [31]. This explains why in the case of patients suffering from COVID-19, the correlation we obtained is rather weak (R = −0.33, *p* = 0.16), since not all of our patients had bacterial infections. Nevertheless, the tendency to change the filterability of RBCs with a change in this indicator indicates that with an increase in inflammation (an increase in the level of procalcitonin), the filterability of RBCs worsens. The concentration of fibrinogen in our study practically did not correlate with the filterability of patients’ RBCs. It was not possible to detect a correlation between filterability and erythrocyte sedimentation rate, but in this case, we attribute this to a very small sample size (n = 5), so it is impossible to make a reliable conclusion about the existence of this correlation. 

Thus, although the data obtained on the effect of inflammation on the filterability of RBCs are clearly insufficient, we may believe, that our assumption that in COVID-19 patients RBCs are damaged as a result of pro-inflammatory mediators’ actions during passing through the foci of the inflammatory process, may be true. This assumption is based on a reliable negative correlation of filterability with the concentration of C-reactive protein, and on taking into account trends, which were demonstrated by such indicators of inflammation as the concentrations of procalcitonin and ferritin.

Summing up, we can say that the present work demonstrates the clinical usefulness of the method for measuring the filterability of erythrocytes to determine the severity of the patient’s condition and the possible outcome of the disease. This method also allows us to investigate the mechanisms underlying the change in the properties of red blood cells in COVID-19 and other pathologies.

## Figures and Tables

**Table 1:** Demographic composition and some parameters of patients’ condition *.

Parameter	Patients (Units)	Parameter (Units)	Patients	Normal Range
n	149	RBC (×10^12^ cells/L)	4.03 (3.41; 4.57)	4.00–5.20 (F) 4.10–5.60 (M)
Sex:		WBC (×10^9^cells/L)	10.09 (6.71; 14.00)	4.00–9.00
		PLT (×10^9^ cells/L)	210.0 (141.0; 294.5)	180.0–320.0
F M	n = 68 n = 81			
		Hct (%)	36.7 (31.0; 41.0)	36.0–49.0 (F) 38.0–54.0 (M)
Age	64 (55; 73) (years)	Hb (g/dL)	11.8 (9.3; 13.4)	12.0–15.0 (F) 13.0–16.0 (M)
		SpO_2_/FiO_2_	265.7 (130.7–352.7)	>452
Weight (on admission)	88 (80; 100) (kg)	MCV (fL)	90.0 (86.4; 93.9)	80.0–100.0
Patient’s condition on admission:		MCH (pg/cell)	29.6 (28.3; 30.8)	27.0–35.0
		MCHC (g/L)	328.0 (321.0; 337.0)	300.0–380.0
		RDW-SD (fL)	46.7 (42.5; 52.1)	35.0–58.0
Moderate Severe Critically severe	n = 58 n = 91 n = 0			
Additional oxygenation:		ALB (g/L)	30.1 (25.8; 33.7)	35.0–52.0
		PRO (g/L)	57.5 (51.4; 63.6)	66.0–83.0
Missing (independent breathing, air, 21% oxygen)	n = 27	CRP (mg/L)	66.92 (16.19; 141.20)	0.00–5.00
		Fng (g/L)	4.65 (3.38; 6.22)	2.00–3.93
Low-flow (fraction of inhaled oxygen 24–35%)	n = 73	APTT (s)	32.7 (28.3; 39.3)	25.1–36.5
		PT (s)	12.9 (12.0; 14.2)	9.4–12.5
High-flow (fraction of inhaled oxygen 40–100%)	n = 48	INR	1.12 (1.04; 1.24)	0.90–1.20
		TT (s)	15.9 (14.3; 19.2)	11.0–20.0
Invasive lung ventilation (oxygen fraction 25–100%)	n = 33	DD ng/mL	2134 (1105; 6176)	0–500
Outcome:		Vs (in TD) (μm/min)	13.5 (8.0; 23.7)	20.0–29.0
Recovery Death	n = 80 n = 69	Vi (in TD) (μm/min)	45.1 (31.2; 54.2)	38.0–56.0

* Averages are represented as medians and ranges (25 to 75 percentiles). Abbreviations used: F—female, M—male; RBC, WBC and PLT—count of erythrocytes, white blood cells and platelets, respectively; Hct—hematocrit; Hb—concentration of hemoglobin; SpO_2_/FiO_2_—the ratio of the percentage of oxyhemoglobin in the blood to the proportion of oxygen in the inhaled air; MCV—mean cell volume; MCH and MCHC—mean content and mean concentration of hemoglobin in erythrocyte, respectively; RDW-SD—red cell volume distribution width (as SD for mean cell volume); ALB—albumin; PRO—total protein; CRP—C-reactive protein; Fng—fibrinogen; APTT—activated partial thromboplastin time; PT—prothrombin time; INR—International Normalized Ratio; TT—thrombin time; DD—D-dimer; TD—test of thrombodynamics; Vs—a stationary rate of clot growth in space; Vi—an initial clot growth rate in space.

## Data Availability

Data presented in this study are contained within the article or Supplementary Materials and are available on request from the corresponding author.

## Supplementary Materials

The following supporting information can be downloaded at: https://www.mdpi.com/article/10.3390/biom12060782/s1, containing Figure S1: Dependence of the erythrocyte filterability on the severity of the patient’s condition taking into account all repeated measurements for each patient, with each represented point colored automatically according to the SpO_2_/FiO_2_ ratio for given measurement; Figure S2: Dependence of the SpO_2_/FiO_2_ ratio value on the severity of the patient’s condition taking into account all repeated measurements for each patient. Figure S3: Dependence of the filterability of erythrocytes on the rate and mechanism of oxygen supply taking into account all repeated measurements for each patient; Figure S4: Dependence of the filterability of erythrocytes on the rate and mechanism of oxygen supply taking into account all repeated measurements for each patient, with each represented point colored automatically according to the SpO_2_/FiO_2_ ratio for given measurement; Table S1: Some additional biochemical and hematological parameters of the patients’ condition.
