# Supplementary material for: Filterability of Erythrocytes in Patients with COVID-19
Source: Biomolecules. 2022 Jun 3;12(6):782. doi: 10.3390/biom12060782 (PMC9220947; doi:10.3390/biom12060782)
Supplement: Supplementary file 1 [file biomolecules-12-00782-s001.zip › biomolecules-1727543-supplementary.pdf]

SUPPLEMENTARY MATERIALS

Dmitry S. Prudinnik, Elena I. Sinauridze, Soslan S. Shakhidzhanov, Elizaveta A. Bovt,

Denis N. Protsenko, Alexander G. Rumyantsev, and Fazoil I. Ataullakhanov

Filterability of erythrocytes in patients with COVID-19

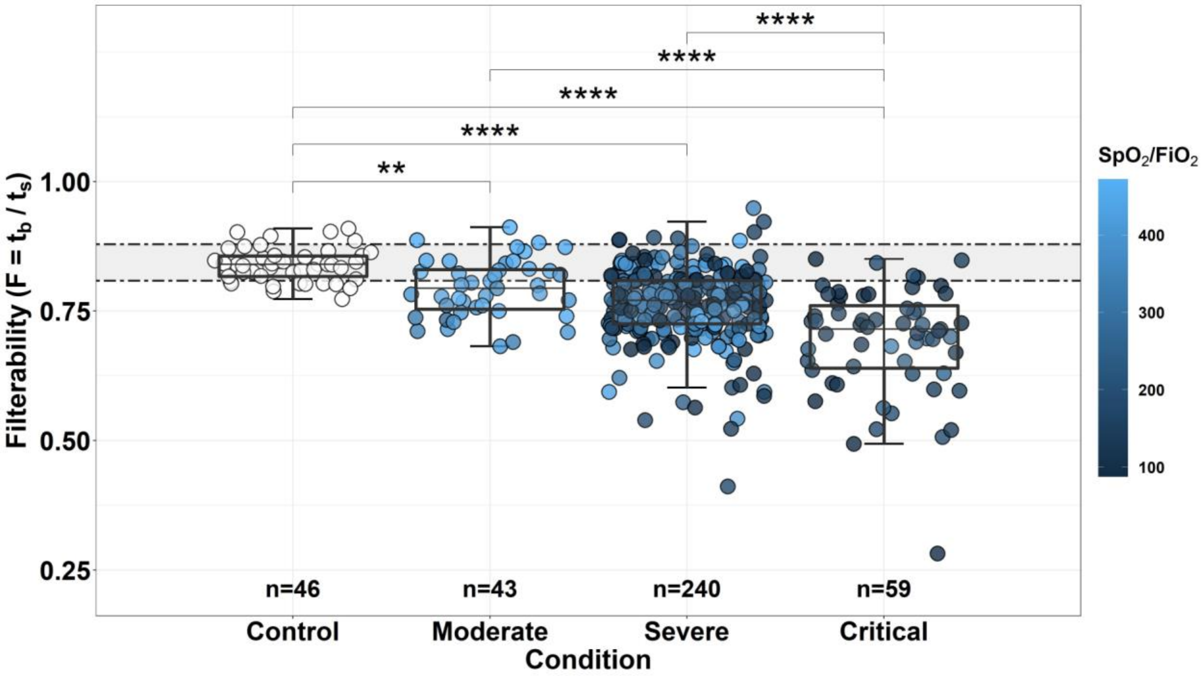

Figure S1. Dependence of the erythrocyte filterability on the severity of the patient's condition taking into account all repeated measurements for each patient. Control - control group, Moderate - a condition of moderate severity, Severe - a serious condition, Critical - a condition of extreme severity. In addition, each represented point is automatically colored according to the SpO_2/FiO_2 ratio for given measurement (see the color scale to the right of the figure). The significance of the differences between the groups was calculated using the Kruskal-Wallis H-test and post-hoc Dunn's test with Bonferroni correction. Horizontal dotted lines show the boundaries of normal filterability values. The box sizes correspond to the range comprising from 25 to 75 percentiles of all measured values. Medians are indicated by horizontal lines, and the length of the whiskers corresponds to the 1.5 interquartile range. The p values for the significance of the differences are shown in figure: ** $p<0.01$ and **** $p<0.0001$.

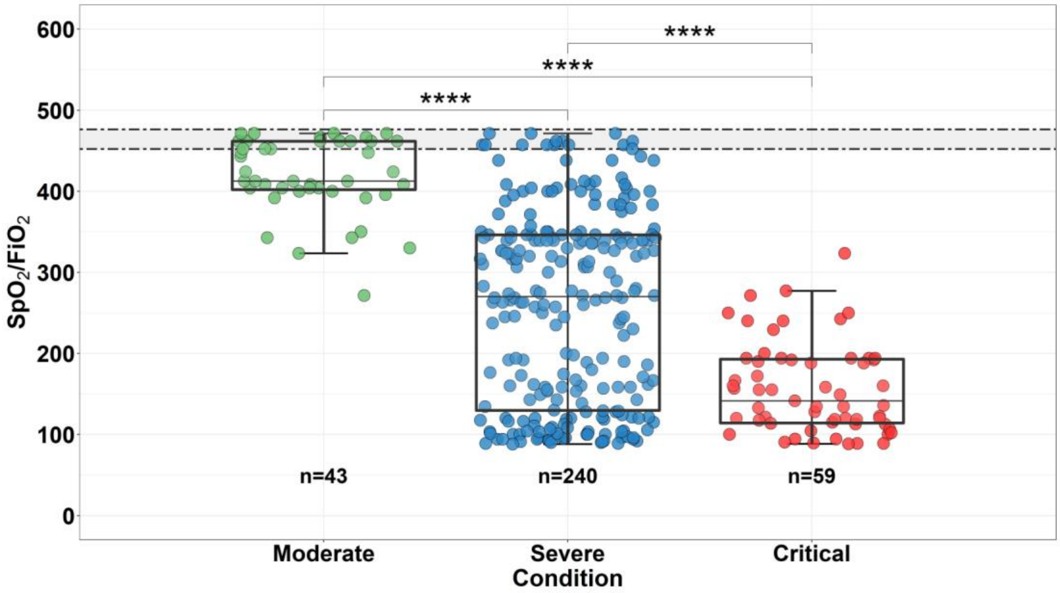

Figure S2. Dependence of the SpO_2/FiO_2 ratio value on the severity of the patient's condition taking into account all repeated measurements for each patient. Moderate - a condition of moderate severity, Severe - a serious condition, Critical - a condition of extreme severity. The control group is not presented, since the values of the SpO_2/FiO_2 ratio were not measured for these donors, but it is recognized that the normal range for these values is > 452. The significance of the differences between the groups was calculated using the Kruskal-Wallis H-test and post-hoc Dunn's test with Bonferroni correction. Horizontal dotted lines show the boundaries of normal values of SpO_2/FiO_2 ratio. The box sizes correspond to the range comprising from 25 to 75 percentiles of all measured values. Medians are indicated by horizontal lines, and the length of the whiskers corresponds to the 1.5 interquartile range. The *p* values for the significance of the differences are shown in figure: **** $p<0.0001$.

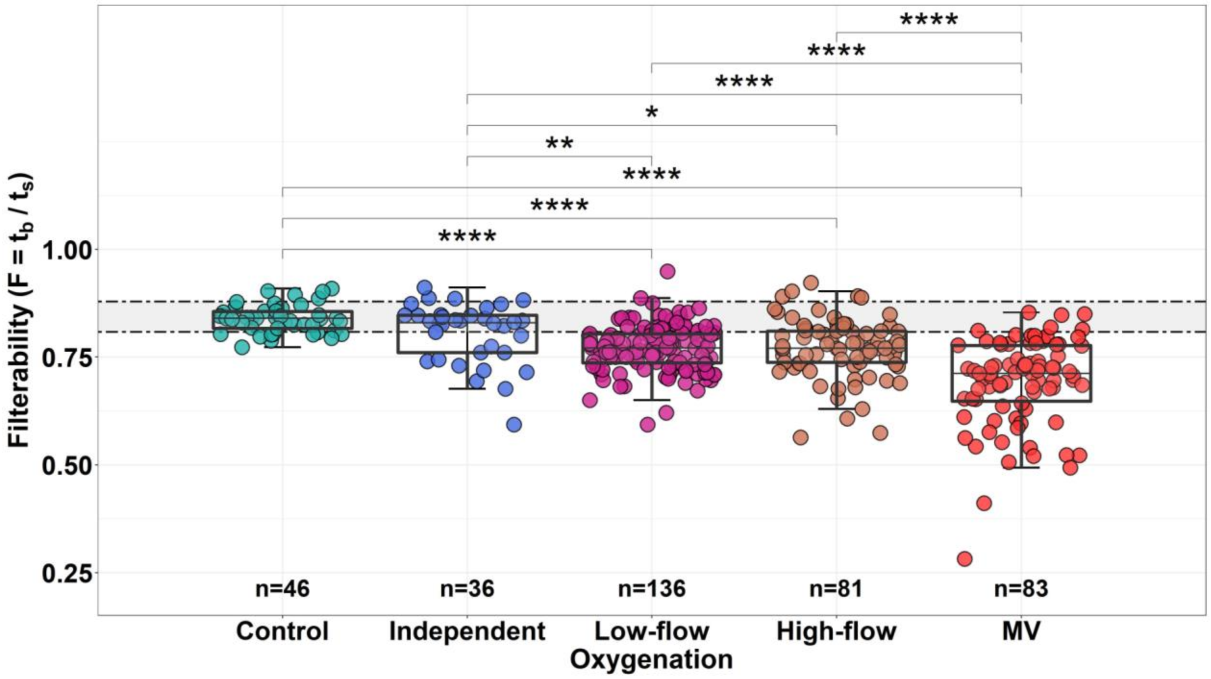

Figure S3. Dependence of the filterability of erythrocytes on the rate and mechanism of oxygen supply taking into account all repeated measurements for each patient. Control (a control group of healthy donors) and Independent (patients without additional oxygen, who breathe with atmospheric air on their own) - oxygen fraction 21%; Low-flow (patients on low-flow oxygenation) - the fraction of oxygen in the inhaled air is 24-35%; High-flow (patients on high-flow oxygenation) - the fraction of oxygen in the inhaled air is 40-100%; MV (patients on invasive mechanical ventilation) - the fraction of oxygen in the inhaled air can be 25-100%. The significance of differences between groups was calculated by the Kruskal-Wallis H-test and post-hoc Dunn's test with Bonferroni correction. Horizontal dotted lines show the boundaries of the normal filterability values. The box sizes correspond to the range comprising from 25 to 75 percentiles of all measured values, and the length of the whiskers corresponds to the 1.5 interquartile range. The p values of the significance of the differences between the groups are shown in the figure: * p<0.05; ** p<0.01 and **** p<0.0001.

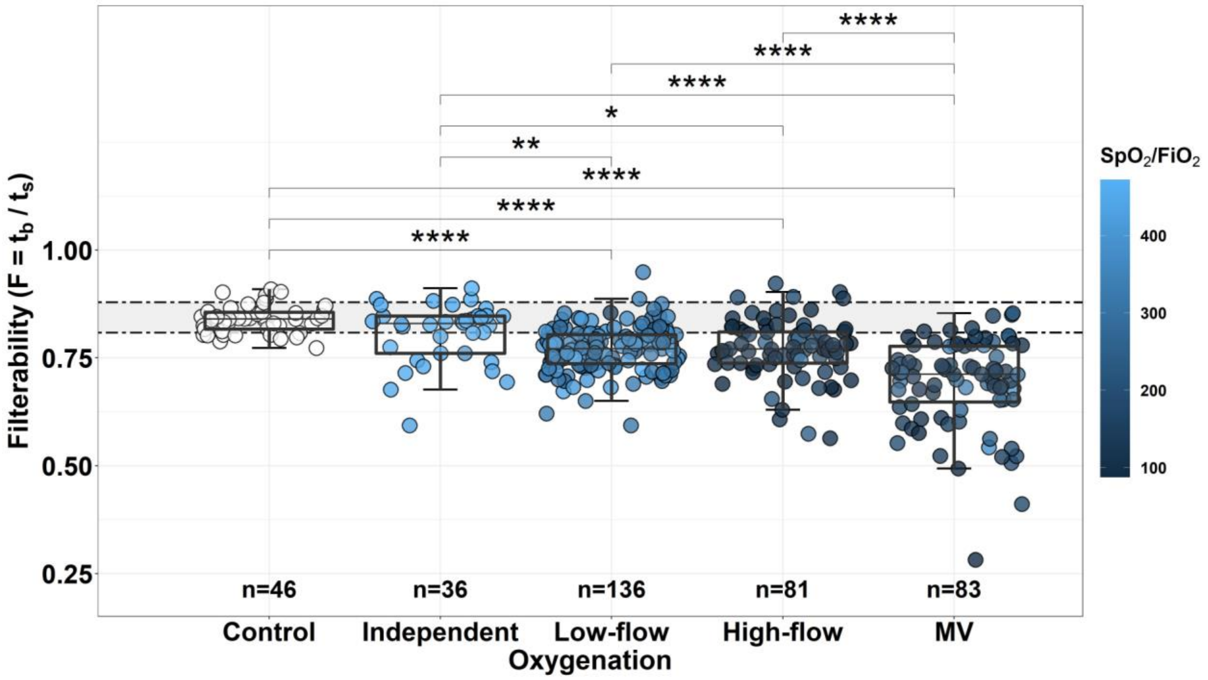

Figure S4. Dependence of the filterability of erythrocytes on the rate and mechanism of oxygen supply taking into account all repeated measurements for each patient. In addition, each represented point is automatically colored according to the SpO_2/FiO_2 ratio for given measurement (see the color scale to the right of the figure). Control (a control group of healthy donors) and Independent (patients without additional oxygen, who breathe with atmospheric air on their own) - oxygen fraction 21%; Low-flow (patients on low-flow oxygenation) - the fraction of oxygen in the inhaled air is 24-35%; High-flow (patients on high-flow oxygenation) - the fraction of oxygen in the inhaled air is 40-100%; MV (patients on invasive mechanical ventilation) - the fraction of oxygen in the inhaled air can be 25-100%. The significance of differences between groups was calculated by the Kruskal-Wallis H-test and post-hoc Dunn's test with Bonferroni correction. Horizontal dotted lines show the boundaries of the normal filterability values. The box sizes correspond to the range comprising from 25 to 75 percentiles of all measured values, and the length of the whiskers corresponds to the 1.5 interquartile range. The p values of the significance of the differences between the groups are shown in the figure: * $p<0.05$; ** $p<0.01$ and **** $p<0.0001$.

Table S1. Some additional biochemical and hematological parameters of the patients' condition.

Parameter, units	Patients*	Normal range
Alanine aminotransferase (U/L)	36.0 (22.0; 63.0)	0.0 – 50.0
Aspartate aminotransferase (U/L)	39.0 (26.0; 66.5)	0.0 – 50.0
pH of blood	7.40 (7.30; 7.45)	7.35 - 7.45
Na^+ iones (mmol/L)	138.6 (136.0; 141.7)	136.0 - 146.0
K^+ iones (mmol/Ll)	3.8 (3.5; 4.3)	3.4 - 4.5
Lactate (mmol/L)	1.5 (1.1; 2.0)	0.9 – 1.7
Glucose (mmol/L)	7.5 (5.9; 10.1)	3. 9 - 5.8
Procalcitonine (ng/mL)	0.37 (0.13; 1.41)	0.00 - 0.50
Creatinine (μmol/L)	82.0 (65.5; 111.0)	49.0 – 104.0
Creatine kinase (U/L)	71.0 (37.0; 212.5)	0.0 – 171.0
Bilirubin total (μmol/L)	11.7 (8.4; 15.9)	5.0 – 21.0
Bilirubin direct (μmol/L)	3.2 (1.9; 5.5)	0.0 – 3.4
Urea (mmol/L)	8.6 (6.1; 13.0)	2.0 - 7.2
Ferritin (ng/mL)	731 (401; 1388)	11 – 150 (Female)<br>20 – 250 (Male)
Erythrocyte sedimentation rate according Panchenkov (ESR)) (mm/h)	24 (9; 43)	2-15
Serum $Fe^{+(2-3)}$ iones (μmol/L)	8.3 (6.0; 12.1)	10.7 – 32.2
Ca^{+2} iones (mmol/L)	1.07 (0.85; 1.19)	3.40 – 4.50
Cl^- iones (mmol/L)	102.0 (98.8; 106.0)	98.0 – 109.0
Standard bicarbonate (mmol/L)	24.3 (20.6; 27.7)	23.0-29.0
PDW-CV (%)**	13.2 (11.7; 15.7)	10.0 – 18.0
Neutrophils ($\times 10^9$, cells/L)	7.30 (4.46; 10.79)	1.56 – 6.13
Mean platelet volume,(fL)	10.9 (9.9; 11.6)	9.4 – 12.4
Eosinophiles ($\times 10^9$, cells/L)	0.01 (0.00; 0.10)	0.04 – 0.36
Lymphocytes ($\times 10^9$, cells/L)	0.94 (0.62; 1.36)	0.76 – 3.33
Basophiles ($\times 10^9$, cells/L)	0.00 (0.00; 0.04)	0.01 – 0.08
Segmented neutrophils (%)	80 (74; 83)	47-72
Stab neutrophils (%)	6 (5; 8)	1-6
Monocytes ($\times 10^9$, cells/L)	0.43 (0.26; 0.68)]	0.12 – 0.99

* Averages are represented as medians and ranges (25 to 75 percentiles).

** PDW-CV, Platelets distribution width by volume (as coefficient of variation), %.
